# The EHA Research Roadmap: Infections in Hematology

**DOI:** 10.1097/HS9.0000000000000662

**Published:** 2021-12-02

**Authors:** Catherine Cordonnier, Per Ljungman, Simone Cesaro, Hans H. Hirsch, Georg Maschmeyer, Marie von Lilienfeld-Toal, Maria Vehreschild, Malgorzata Mikulska, Marieke Emonts, Andrew R. Gennery, Dionysios Neofytos, Pierre-Yves Bochud, Hermann Einsele, Johan Maertens

**Affiliations:** 1 University Paris Est-Créteil, France; 2 Division of Hematology, Department of Medicine Huddinge, Karolinska Institutet, Stockholm, Sweden; 3 Department of Cellular Therapy and Allogeneic Stem Cell Transplantation, Karolinska Comprehensive Cancer Center, Karolinska University Hospital Huddinge, Stockholm, Sweden; 4 Pediatric Hematology Oncology, Department of Mother and Child, Azienda Ospedaliera Universitaria Integrata, Verona, Italy; 5 Transplantation & Clinical Virology, Department Biomedicine, University of Basel, Switzerland; 6 Clinical Virology, Laboratory Medicine, University Hospital Basel, Switzerland; 7 Infectious Diseases & Hospital Epidemiology, University Hospital Basel, Switzerland; 8 Department of Hematology, Oncology and Palliative Care, Klinikum Ernst von Bergmann, Academic Teaching Hospital of Charité University Medical School, Berlin, Germany; 9 Universitätsklinikum Jena, Klinik für Innere Medizin II, Jena, Germany; 10 Department of Internal Medicine, Infectious Diseases, University Hospital Frankfurt, Goethe University Frankfurt, Germany; 11 Division of Infectious Diseases, University of Genoa (DISSAL), Italy; 12 IRCCS Ospedale Policlinico San Martino, Genova, Italy; 13 Paediatric Immunology, Infectious Diseases & Allergy, Great North Children’s Hospital, Newcastle upon Tyne Hospitals NHS Foundation Trust, United Kingdom; 14 Translational and Clinical Research Institute, Newcastle University, Newcastle upon Tyne, United Kingdom; 15 Paediatric Immunology and HSCT Translational and Clinical Research Institute Newcastle University Great North Children’s Hospital Newcastle upon Tyne, United Kingdom; 16 Service des Maladies Infectieuses, Hôpitaux Universitaires de Genève, Switzerland; 17 Service des maladies infectieuses, Département de médecine, Centre hospitalier Universitaire Vaudois et Université de Lausanne, Switzerland; 18 Department of Internal Medicine II, University Hospital Würzburg, Germany; 19 Universitaire Ziekenhuizen Leuven, Belgium

*In 2016, the European Hematology Association (EHA) published the EHA Roadmap for European Hematology Research*^1^ *aiming to highlight achievements in the diagnostics and treatment of blood disorders and to better inform European policymakers and other stakeholders about the urgent clinical and scientific needs and priorities in the field of hematology. Each section was coordinated by 1 to 2 section editors who were leading international experts in the field. In the 5 years that have followed, advances in the field of hematology have been plentiful. As such, EHA is pleased to present an updated Research Roadmap, now including 11 sections, each of which will be published separately. The updated EHA Research Roadmap identifies the most urgent priorities in hematology research and clinical science, therefore supporting a more informed, focused, and ideally a more funded future for European hematology research. The 11 EHA Research Roadmap sections include Normal Hematopoiesis; Malignant Lymphoid Diseases; Malignant Myeloid Diseases; Anemias and Related Diseases; Platelet Disorders; Blood Coagulation and Hemostatic Disorders; Transfusion Medicine; Infections in Hematology; Hematopoietic Stem Cell Transplantation; CAR-T and Other Cell-based Immune Therapies; and Gene Therapy.*

The authors dedicate this section to the memory of the late Professor Claudio Viscoli, who contributed as a co-author on the subsection “Non-neutropenic hematology patients including those treated with new targeted therapies.” Claudio Viscoli, who was an infectious diseases specialist, has been a pioneer in the understanding and management of infections in immunocompromised hosts, especially hematology patients. Being a leader of major international initiatives and achievements, Claudio was a mentor and a constant inspiration for many infectious diseases specialists and hematologists and has vigorously encouraged clinical research in these patient populations.

Since the initial publication of the EHA Roadmap, major advances have been achieved in the management of infectious complications in the hematology ward. One of the best examples is the development of letermovir for the prophylaxis of cytomegalovirus (CMV) reactivation in seropositive allogeneic hematopoietic stem cell transplantation (HSCT) recipients. Concomitantly, many new therapies have been developed for various hematologic malignancies, providing new infectious risks. Europe as a whole and many national societies have highly contributed to research in this field, including but not limited to large groups such as the European Group for Blood and Marrow Transplantation (EBMT), the European Organization for Research and Treatment of Cancer, and the European Society of Clinical Microbiology and Infectious Diseases. Also, over the last 15 years, the European Conference on Infection in Leukaemia (ECIL) has elaborated specific guidelines for diagnosis, prevention, and treatment on the main infectious complications in patients with hematological disorders. In addition, an EHA Scientific Working Group on Infections in Hematology was installed in 2017. Despite all this progress, much remains to be done to understand the mechanisms leading to infectious complications in hematologic malignancies and to develop pertinent strategies to overcome or decrease the risks. Also, the ongoing severe acute respiratory syndrome coronavirus 2 (SARS-CoV-2) pandemic has emphasized the need for national and international collaboration using big data, computer modeling, and machine-learning models for evidence-informed medicine.

## Infections in HSCT recipients

### Introduction

Infections have been major obstacles to the success of allogeneic stem cell transplantation. Transplant strategies are continuously evolving with the introduction of new conditioning regimens and an increased utilization of haploidentical donors. Many of these newer approaches will affect the dynamics, sequence, and degree of immune reconstitution as well as impact the risk of infections. In this field, knowledge of the risk of specific infections in short-, intermediate-, and long-term perspectives is still incomplete.

Viral infections have been recognized as important for outcome, especially in allogeneic stem cell transplant recipients. The introduction of the new antiviral drug letermovir has reduced morbidity and mortality of CMV.^2,3^ Despite this, patients still develop severe CMV disease, although less frequently. Adenovirus infections have emerged as important threats, especially in high-risk pediatric transplantation. It has been difficult to develop effective and safe antiviral treatments.^4,5^ Similarly, BK polyomavirus remains a significant unmet clinical need for hemorrhagic cystitis that is re-emerging post-transplant with recent types of conditioning regimens.^6^ Adoptive immunotherapy with virus-specific lymphocytes is still considered experimental and limited to academic groups or clinical research projects. Its applicability on a broader scale requires more data as well as cellular products easily accessible and affordable.

A changing epidemiology of viral infections poses new challenges. The ongoing pandemic with SARS-CoV-2 causing coronavirus disease 2019 (COVID-19) shows this extremely clearly. During the past decades, at least 10 other new respiratory viruses have been described and the yearly influenza pandemic remains a potential risk.^7^ Similarly, West Nile virus, Chikungunya virus, Zika virus, and hepatitis E virus have emerged as potential threats. Several of these novel viruses have also the potential to be transmitted from stem cell donors, possibly requiring testing in routine donor evaluations. Multidrug-resistant (MDR) bacterial infections are major challenges in some countries and the risk for spreading is of international concern. Fungal infections also remain as a threat, especially from difficult to treat species such as Mucorales and the recently emerged *Candida auris*. Vaccines are important for preventing infections in the general population and in close contact persons to stem cell transplant recipients. There remain major gaps in our knowledge of how best to utilize vaccines in stem cell transplant recipients.^8^ The emerging insight into the essential role of the human microbiome and the gut-immune axis in immunocompromised patients, including allogeneic HSCT recipients, is highlighted in the chapter on “infections in neutropenic patients.”

### European research contributions

European centers and collaborative groups, such as the Infectious Diseases Working Party of the EBMT, have important roles. Topics of particular interest for European investigators include the management of viral infections, the development of vaccination strategies for HSCT recipients, and diagnosis and management of fungal and MDR bacterial infections. Many of these studies have changed practice, not only in Europe.

### Proposed research for the Roadmap

- Markers and dynamics of immune reconstitution with emphasis on new transplant strategies and specific populations:
  a) Haploidentical transplantation
  b) Elderly patients (>60 y, 65 y, 70 y, or others?)
  c) Pediatric patients
- Viruses.
  a) CMV: Strategies are needed for evaluation of new prophylactic and therapeutic modalities and their (detrimental or protective?) impact on graft-versus-malignancy and graft-versus-host disease.
  b) Adenovirus: Strategies for management including evaluation of new antiviral agents and adoptive T-cell therapy.
  c) BK polyomavirus: Strategies for conditioning, immunosuppression, antivirals, antibodies, T-cell therapy.
  d) Respiratory viruses: Identification and surveillance of respiratory viruses including SARS-CoV-2 in allogeneic HSCT to establish preventive strategies including infection control, vaccines, and effective antiviral therapy.
- Bacterial and fungal infections.
  a) Antibiotic stewardship to decrease the risk of MDR bacteria should be encouraged.
  b) Controlled clinical studies in difficult-to-treat and difficult-to-diagnose fungal infections are needed.
- T-cell therapy for infections.
  a) Approaches to improve the immune reconstitution with a minimal risk of uncontrollable graft-versus-host disease.
  b) Multispecific T cells with activity against several infections, especially for prevention of viral infections.
  c) Virus-specific T cells need to be evaluated in well-designed controlled trials.
- Vaccines.

The following topics need to be addressed:

a) Different vaccine schedules should be used in patients having undergone different transplant procedures.
b) New vaccines, such as the inactivated varicella zoster vaccine, CMV vaccines, and vaccines against SARS-CoV-2, should be evaluated in well-designed studies.
c) Study of the humoral and T-cell immune responses following vaccination and exploring differences in reactogenicity between heterologous and homologous schedules.

### Anticipated impact of the research

In past decades, major improvements have been achieved in infectious disease management in allogeneic HSCT recipients. Despite these advances, infections remain important causes of nonrelapse mortality. New infections can quickly become severe threats, as shown by the ongoing COVID-19 pandemic. Therefore, the impact of the proposed projects will be in 2 areas: to further reduce the morbidity and mortality of infections and to develop management strategies that quickly can meet new challenges.

## Infections in neutropenic patients

### Introduction

Infections are the leading cause of death in patients with hematologic malignancies undergoing myelosuppressive chemotherapy. The majority of infections are bacterial, invasive fungal, or viral infections.

### Proposed research for the Roadmap

The most pressing medical and scientific needs that remain unmet in the field of infections in neutropenic patients include (1) the need for antimicrobial stewardship in the face of emerging MDR bacteria given the lack of new antimicrobial agents, (2) the challenge of community-acquired respiratory viruses, (3) improvements in infection control, and (4) establishing the role of the microbiome.

- Antimicrobial stewardship in prophylaxis and therapy. The current standard of care for neutropenic patients is a predefined form of antimicrobial prophylaxis and empiric antimicrobial therapy as soon as fever occurs. This has been a lifesaving approach for many years, but several issues remain: first is improving the initial response to antimicrobial therapy, second is establishing the minimum duration of systemic antimicrobial therapy after defervescence, as current approaches often result in unnecessarily prolonged use of antibiotics. Thirdly, in the era of MDR pathogens,^9^ the current recommendations for antimicrobial prophylaxis and treatment even after establishing an etiology needs to be reevaluated. Thus, we propose research regarding the following topics:
  ✓ Use of therapeutic drug monitoring to personalize dosing and improve response for prophylaxis and initial empirical therapy
  ✓ Prospective comparison of the duration of antibiotic treatment, including in de-escalation (step-down) strategies, with or without guidance by inflammatory markers such as C-reactive protein, procalcitonin, interleukin-6, and markers of gut function (eg, citrulline)
  ✓ Prospective evaluation of different antimicrobial prophylaxis regimens utilizing commonly used fluoroquinolones, as well as “older” drugs such as cotrimoxazole with prospective monitoring of inflammatory markers and citrulline
  ✓ Prospective evaluation of a systematic approach based on clinical and laboratory findings to guide preemptive compared with empiric antimicrobial therapy including antifungal therapy
  ✓ Clinical trials of novel antibacterial agents with new targets and mechanisms of action in neutropenic patients (novel drugs as well as bacteriophages)
- Community respiratory viruses (CRV) excluding SARS-CoV-2. Over the past decade, local outbreaks have brought CRVs to the attention of clinicians and scientists.^10^ CRVs are regarded as an uncommon cause of fever during neutropenia, they tend to occur seasonally and are difficult to diagnose, but they have not yet been studied systematically. However, the increasing number of reports of fatal CRV infections illustrates the need for better understanding of CRV epidemiology. Further research is warranted, and we suggest the following topics for clinical studies:
  ✓ Studies on epidemiology, seasonality, and clinical relevance of CRVs (other than SARS-CoV-2) in neutropenic patients using polymerase chain reaction (PCR)-based platforms and rapid point of care tests
  ✓ Prospective evaluation of infection control measures, especially in asymptomatic patients shedding the virus
  ✓ Clinical development of novel antiviral agents with activity against relevant CRVs.
- Efficacy of infection control measures. Infection control measures have been adopted routinely to reduce the risk of hospital-acquired infections. These measures include protective isolation, use of gloves and gowns, rigorous disinfection measures, reduction of inhaled potentially infective particles by air filtration and masks worn by patients leaving their rooms or wards, and low-microbial hospital diets. The evidence of benefit from these measures in terms of reducing infection-related morbidity and mortality is lacking.^11^ In light of an increasing threat from MDR bacteria and fungi among hematologic patients, a critical reappraisal from prospective, randomized clinical studies focused on the use of the following is needed:
  ✓ Use of low-microbial hospital diet
  ✓ Benefit from high-efficiency particulate air (HEPA) filtration
  ✓ Rationale of disposable gowns, gloves, and masks for caregivers and visitors and of well-fitting masks for patients outside their treatment rooms
- Microbiome. In recent years, a rapidly evolving insight into the essential role of the human microbiome for the quality of immune responses in normal and immunocompromised individuals has been attained.^12^ The potential impact of preserved gut microbiota diversity on patient outcome has been supported by data from allogeneic stem cell transplant recipients and patients undergoing checkpoint inhibition treatment.^13,14^ At the example of these patients, it has become clear that a deeper understanding of the gut-immune axis in the context of hematological diseases may revolutionize future treatment strategies. In addition, recent findings have revealed microbiota dynamics preceding development of infections with MDR pathogens.^15,16^ There is a need to translate novel insights into the role of the human microbiota in immune modulation and infection pathogenesis into preventive and therapeutic options. Clinical and translational studies in hematological patients are required to explore the following aspects:
  ✓ Prediction of treatment outcome and infectious complications in specific populations
  ✓ Acquisition of a functional understanding of the gut-immune axis
  ✓ Identification and isolation of key microbiota and/or their metabolites
  ✓ Development of preventive and/or therapeutic microbiota-based drugs and strategies.

### Anticipated impact of the research

These projects will result in the following:

✓ A significant improvement of survival rates of hematological patients through an enhanced antitumor response and a reduction of infectious complications
✓ An improvement of the quality of life in hematological patients through maintenance of their physiological microbiota
✓ Reduction of unnecessary antimicrobial prophylaxis and therapy leading to reduced pathogen and commensal resistance rates
✓ Evidence-based update of the guidelines on the rational use of infection control measures for severely immunocompromised patients
✓ A fundamental revision of the current approach to fever and infections in neutropenic patients.

## Non-neutropenic hematology patients including those treated with new targeted therapies

### Introduction

Over the last 5 years, important progress in studying and understanding the importance of infectious complications in patients with hematological malignancies treated with various targeted agents has been made. Several scientific associations, including ESCMID and ECIL, have dedicated specific papers to this issue and highlighted particular associations with different targeted therapies.^17,18^

One of the main findings on this issue includes discovering increased rates of infectious complications. Here, mainly viral reactivations and fungal infections were reported following the use of new drugs (eg, idelalisib) or combinations of drugs (eg, rituximab and bendamustine) designed initially for more fragile patients. New treatments such as ibrutinib have been associated with an increased risk for invasive aspergillosis (IA) of the central nervous system (CNS). For other targeted treatments, uncertainties remain on the risk of infectious complications and their optimal management.

Furthermore, the main criteria in the diagnosis of fungal infections in immune-compromised patients with hematological malignancies have confirmed the role of molecular diagnostics in this setting. This is important since serum fungal antigens have low diagnostic yield in non-neutropenic subjects.^19^

Updated guidelines on vaccination in patients with hematological malignancies have been published.^20^ This work highlighted the paucity of the data on the efficacy of selected vaccines in this population (eg, anti-meningococcal, human papillomavirus outside the approved age limits) and the challenge of optimal timing in those with long-lasting treatments. Several problems associated with viral hepatitis have changed during the last years. Given the overall success of treatment for hepatitis C virus (HCV) infection with directly acting agents (DAAs), the management of this infection shifted towards almost universal treatment (potentially excluding patients receiving palliative care only). Although the relevant hepatology guidelines suggest the use of high barrier drugs even for prophylaxis of hepatitis B virus (HBV) reactivation in those with past HBV infection, there are little data for lymphoma patients treated with chemotherapy including anti-CD-20 antibodies, and for many novel drugs administered life-long (eg, dasatinib), the cost-effectiveness of prophylaxis vs. monitoring needs to be established. Also, in certain geographical areas, there is an increased risk of hepatitis E viral infection, which may become chronic in immunocompromised patients; thus, maximum awareness is required for early diagnosis and potential treatments.

Finally, the pandemic spread of SARS-CoV-2 infection has put an enormous strain on this population, which is considered at high risk for acquiring the disease and for death, but in whom limited data are available on the optimal management strategies for both the underlying disease and the SARS-CoV-2 infection, including the treatment of the inflammatory phase. Few positive changes brought in by this pandemic are the emphasis on the relevance of daily preventive measures, including personal protective equipment and the need for social distancing and quarantine, the importance of immunization, and an increased use of telemedicine that allows to reduce the physical contact with healthcare structures.

### Proposed research for the Roadmap and anticipated impact

- Evaluation of the infectious risk (ie, incidences, presentations, etiologies) in case of sequential therapy that has become the standard of care in many chronic hematological malignancies
- Studies on the impact of effective HCV eradication with DAAs and the cost-effectiveness of long-term HBV reactivation prophylaxis
- Studies on the impact of multidrug-resistant pathogens in non-neutropenic hematology patients and the challenges of implementing effective antimicrobial stewardship in this population
- Studies on the optimal timing and dose schedule of various vaccinations, including those against SARS-CoV-2. In addition, studying the humoral and T-cell immune responses following vaccination, understanding the lack of immune response on some of these patients (eg, chronic lymphocytic leukemia) and exploring differences in reactogenicity between heterologous and homologous schedules.
- Development of rapid point of care tests for more effective diagnosis (eg, effective antigen tests to detect different respiratory viruses, including SARS-CoV-2 and influenza; lateral flow assays for fungal infections)
- Long-distance patient monitoring through telemedicine, which may provide the opportunity for routine collaboration with highly specialized centers without the need for patient travel

## Infections in primary immune deficiencies

### Introduction

Although individually rare, there are now over 450 genetic inborn errors of immunity described, double the number from 2015.^21^ The lifetime infection burden in this group is high; infections can be unusual and difficult to treat. The risk is increased by added immunosuppression required for treatment.

Early diagnostics and effective treatment for bacterial, viral, and fungal infection remain important.^1^ The research is relevant for various patient populations, with different susceptibility to infections depending on the type of immunodeficiency.

### European research contributions

International collaboration including next generation sequencing, introduction of newborn screening, and international guidelines have facilitated diagnosis of immunodeficiencies in increasing numbers of patients. Diverse phenotypes with the same genetic disorders are increasingly identified. Viral PCR tests allow more rapid accurate diagnosis; furthermore, new antiviral and antifungal drugs improve outcomes, although accessibility of these drugs remains a challenge. Representation of clinicians and scientists in the European Society for Paediatric Infectious Diseases, ESCMID, and the European Society for Immunodeficiencies improves the collaborations.

### Proposed research for the Roadmap

- Viral infections. PCR allows accurate identification and quantification of many viruses, but for new viruses such as SARS-CoV-2, sensitivity is limited for throat swabs that are commonly used. Promising developments in therapeutic options have become available in adults, with few in children, although increased toxicity in adults, not observed in children, might result in withdrawal of nephrotoxic drugs like brincidofovir. Induction of drug resistance remains an issue as well as lack of CNS penetration, an important limiting factor in using antivirals active against CMV. Treatment and prevention of respiratory viruses including SARS-CoV-2 remain a major issue. For the latter, there is limited information in immune-compromised patients. With the exception of patients with autoantibodies against type I interferon, risks so far appear largely in line with the general population.^22,23^ Potential for reactivation with stem cell transplant, recurrent infections, and triggering of immunodysregulation are currently being evaluated in large international collaborative efforts.
- Central line-associated bloodstream infections. This remains a significant issue in oncology and chronic diseases. The morbidity and mortality of central line-associated bloodstream infections necessitate all children with fever and central lines to undergo blood culturing and empirical broad-spectrum antibiotic administration that may augment antimicrobial resistance in a high-risk population. Recent efforts have reduced duration of intravenous antibiotics for those with low risk, safely reducing antibiotic exposure and hospital admissions.^24^
- Gastrointestinal infections. Primary immune deficiency (PID) patients harbor many bacterial and viral gut pathogens with an impact on nutrition and fluid management, as well as a significant cause of morbidity and mortality. While much research has examined the effect of the gut microbiome on immune response, no firm conclusions have altered the diagnostic or therapeutic management.
- Infections associated with biologics. Biologic agents, particularly monoclonal antibodies against pro-inflammatory cytokines or receptors, are more widely used, increasing the risk of infections with atypical presentation in a high-risk population (PID but also many other patients with hematological disorders).^25^
- Diagnostic fungal markers and treatment monitoring. Morbidity and mortality related to invasive fungal infections are recognized in PID patients, especially those receiving immunosuppression. Large inter-individual variability of antifungal concentrations observed in children is described in pharmacokinetic studies warranting prompt informed adjustment of dosing, allowing optimal treatment of these potentially lethal infections. Improving absorption of long-term oral therapy is essential.

### Anticipated impact of the research

The impact of these proposed research areas will significantly improve management of PID patients. Vulnerability to common and opportunistic pathogens often results in infections with multiple pathogens, which are not cleared completely until months after HSCT. Determining which pathogen is causing symptoms, rather than simply colonizing, is important to enable safe rationalized treatment approaches with reduced toxicity. Host response-driven diagnostic approaches could be available in the future, although they will need to be carefully looked at for individual immunodeficiencies resulting in a modified response.

## Genetic predisposition factors for infection in hematology and hematopoietic stem cell transplantation patients

### Introduction

Significant progress has been achieved in the field of immunogenetics and infectious diseases over the last 5 years. Additional data have almost exclusively focused on single-nucleotide polymorphisms (SNPs) of genes coding for 2 important pattern recognition receptors, namely pentraxin-3 (PTX3) and *Dectin-1*.

### Pentraxin 3

PTX3 can recognize and bind to *Aspergillus* conidia, facilitate opsonization and subsequently lead to complement and phagocyte activation.^26–28^ Specific PTX3 SNPs have been identified as important risk factors for IA and other invasive mold infections (IMIs) in high-risk patient populations, such as allogeneic HSCT and solid organ transplant recipients.^29–31^ More recently, in a retrospective study on a cohort of acute leukemia patients who—for their vast majority—did not receive primary anti-mold prophylaxis, a trend for more IMI was described for patients carrying the PTX3 h2/h2 haplotype (log-rank test *P* = 0.17).^32^ When patients were stratified according to the presence (N = 82, 36%) or absence (N = 144, 64%) of neutropenia, the association was significant only in those who were not neutropenic (*P* = 0.048). In non-neutropenic patients, the association was even more significant when considering PTX3 SNPs separately (*P* = 0.018 for rs2305619, *P* = 0.017 for rs3816527, both in a recessive mode of inheritance), or when considering patients homozygous for any of them (*P* = 0.005) suggesting that homozygosity for any of these SNPs may be more relevant.

### Dectin-1

Dectin-1 is a transmembrane C-type lectin-like receptor present on myeloid cell surface binding to the beta-glucan component of fungal cell wall and leading to signaling pathway activation for the control of fungal infections.^33^ The rs16910526 (Y238X) SNP was the first to draw attention, with its presence in allogeneic HSCT recipients associated with higher rates of gastrointestinal tract colonization with *Candida* species.^34^ Subsequently, the rs16910526 (Y238X) polymorphism in either donors or recipients of allogeneic HSCT was found to be significantly associated with the risk for IA, particularly when the polymorphism was present in both, donors and recipients, with an adjusted hazard ratio of 3.9 (*P* = 0.005).^35^

### Proposed research for the Roadmap

- To better understand the effect of genetic polymorphisms in donors and/or recipients on the infection risks after allogeneic HSCT, in conjunction with donor-recipient chimerism post-transplant.
- To generate data from patients who receive treatment with immunotherapeutic and molecular targeted agents (such as but not limited to brentuximab vedotin, blinatumomab, cytotoxic T lymphocyte antigen 4 inhibitor, and programmed cell death 1/programmed cell death ligand 1 inhibitor as well as ibrutinib, idelalisib, histone deacetylase inhibitors, mechanistic target of rapamycin inhibitors, ruxolitinib, and venetoclax) and/or chimeric antigen receptor T-cells (CAR-T cells) (see subsection CAR-T) are urgently needed.

### Anticipated impact of the research

The existing literature shows similar associations between PTX-3 or Dectin-1 polymorphisms and IA and IMI in different populations. The high frequency of some alleles in the general population of up to 30% makes PTX3 polymorphisms an important target for future research. These findings require further investigation and could represent the basis for additional interventional clinical trials in the field of diagnosis, targeted antifungal prophylaxis, and treatment in hematology patients. The recent associations between CMV infection, survival and PTX3 polymorphisms are definitely intriguing. However, more data are required to assess the potential associations of PTX3 SNPs and risks for viral, including CMV, infections, and other infections occurring after HSCT, particularly when considering the strong associations between CMV infection and risk for IA in allogeneic HSCT recipients. Finally, although initial data on *Dectin-1* SNP rs16910526 were not subsequently confirmed, it appears that 2 other *Dectin-1* polymorphisms (rs3901533 and rs7309123) may be important targets for future research in high-risk hematological patients.

## Infections after chimeric antigen receptor T-cell therapies

### Introduction

Although the access to CAR-T cell therapy is still limited due to the necessary technical resources of the centers, CAR-T cells can induce durable responses in hematologic malignancies, offering a new treatment for patients who would have been considered some years ago, as being beyond any therapeutic option. However, CAR-T cells are associated with significant complications; at least one third of the patients develop infectious complications, especially during the first months after infusion when still neutropenic.^36^ In a series of 133 consecutive patients, the risk factors for developing infections during the first 3 months were to have an acute lymphoblastic leukemia (ALL), to have received ≥ 4 previous regimens of chemotherapy, and to have received a high dose of CAR-T cells.^36^ Most of these infections were of bacterial origin. Viral infections are rare, herpes virus infections are uncommon under valaciclovir prophylaxis, and thus respiratory virus infections are predominant. It is very likely that in multiple myeloma (MM) patients, the risk of viral infections will be higher due to the more intensive pretreatment including long-term steroids. Fungal infections, including pneumocystosis, are rare especially in MM patients.

The infectious risk after CAR-T cell infusion is multifactorial. Most patients referred for CAR-T cell therapy had received several lines of treatment.^36,37^ Half of them had received HSCT,^38,39^ were lymphopenic and severely immunocompromised before lymphodepletion and CAR-T infusion. Moreover, most of them had already received several courses of antibacterial therapy, so that the level of bacterial resistance was probably high.^37^ As a consequence of the lymphodepleting chemotherapy administered before CAR-T cell infusion, neutropenia is common for up to more than 4 weeks (median duration: 8–12 d). Consistently, 80% of the infections occur within the first 10 days and are of bacterial origin.^36,37^ As the 2 approved CAR-T cell products (tisagenlecleucel and axicabtagene ciloleucel) are both targeting CD19+ B-lineage neoplasms, most experience until now is in acute lymphoblastic leukemia B and other B-cell lymphoproliferative diseases. Unfortunately, this target is not tumor-specific and also affects normal CD19+ B cells, inducing a prolonged B-cell immune deficiency especially in patients with ALL.^40^ Most patients have a major B-cell depletion during the 4 weeks after the infusion. Among those who survived at least 12 months after CAR-T cell infusion having a median follow-up of 28 months, 40% of the patients are still hypogammaglobulinemia or have received intravenous immuneglobulin (IVIG).^41^ Despite the frequency of hypogammaglobulinemia after CD19+-CAR-T therapy, some pathogen-specific, long-term immunity may be preserved, possibly from preexisting long-lived plasma cells that poorly express CD19.^42^

### Proposed research for the Roadmap and anticipated impact

The success of CAR-T cells in hematologic malignancies has also opened new research areas for using CAR-T cells in viral and fungal infections^43^ so that hematology patients can also benefit from this approach for preventing or treating some opportunistic infections. Although cooperative groups have released guidelines for the management of infectious complications in these patients, many issues are unknown and should be explored.^44^ The current data on infectious complications after CAR-T cell therapy suggest the following areas of research for the next future:

✓ Define infusion risk factors for each type of infection, including hypogammaglobulinemia, in order to optimize the choice and timing of drug prophylaxis and IVIG replacement
✓ Explore the relationship between cytokine release syndrome, immune effector cell-associated neurotoxicity syndrome, and infections and how the occurrence of one of these events could impact the other
✓ Investigate the long-term effects of CAR-T therapy on B-cell immune recovery, kinetics of specific antibodies, and vaccine response

**Summary box: Main research & policy priorities**

1. Despite considerable progress over the last decade, infection remains a main cause of death in hematology patients. A specific strategy for each type of the more frequent infections should be elaborated in the hematology centers. The more likely risks should benefit of prophylaxis each time possible.
2. In neutropenic patients, the strategies of deescalation are the most likely to reduce the use of antibacterials in the hematology ward and consequently decrease the risk of resistance.
3. Respiratory virus infections may be life-threatening, especially in stem cell transplant recipients. Epidemiology data and active therapeutics are needed to limit the risk and improve the prognosis.
4. Many targeted drugs are currently assessed for various hematological malignancies. For each of them, it is crucial to collect all infectious data from the early development of the drug, in order to know about the risk they provide for specific infections and to develop a preventative strategy if necessary.
5. Assessing the relationship between genetic polymorphisms and predisposition to certain infections should allow a targeted, individual management, avoiding unnecessary risks of drug toxicity and drug interferences in most patients.

## Disclosures

CC received royalties of payments from MSD, Takeda, and Gilead Science. PL received advisory board member to AiCuris, Takeda, royalties of payments from Takeda, MSD, Pfizer, Gilead, DSMD/DMC/EAC from OctaPharma, Enanta pharmaceuticals, and Takeda. SC received fee for lectures by Gilead Sciences. HHH received consulting for Roche Diagnostics. GM received honoraria for lectures from Gilead, GSK, Merck-Serono, AMGEN, Janssen-Cilag, AstraZeneca, and Bristol-Myers Squibb. MvLT received grants from Gilead, Oncopeptides, Celgene, DFG, Deutsche Krebshilfe, royalties of payments from Celgene, Gilead, Chugai, Janssen, Novartis, Amgen, Takeda, BMS, Medac, Oncopeptides, Merck, CDDF, AbbVie AstraZeneca, Pfizer, 4DPharma, and Shionogi. MV received consulting for Alb Fils Kliniken GmbH, Arderypharm, Bio-Mérieux, DaVolterra, Farmak International Holding GmbH, Ferring, Immunic AG, MaaT Pharma, Roche, SocraTec R&D GmbH, Grants from 3M, Astellas Pharma, Biontech, DaVolterra, Evonik, Gilead Sciences, Glycom, Immunic, MaaT Pharma, Merck/MSD, Organobalance, Seres Therapeutics, Takeda Pharmaceutical, royalties of payments from Astellas Pharma, Basilea, Gilead Sciences, Merck/MSD, Pfizer, and Organobalance. MM received royalties of payments from Pfizer, Gilead, and Janssen. ARG received grants from JAZZ Pharmaceuticals. DN received consulting and grants from MSD and Pfizer. HE received consulting for Janssen, BMS/Celgene, Amgen, Novartis, Takeda, Grants from Janssen, BMS/Celgene, Amgen, Novartis, Takeda, GSK, and Sanofi. JM received consulting for MSD, Gilead Sciences, Pfizer Inc., Astellas Pharma, Takeda, Mundipharma, Cidara, F2G, Scynexis, Grants from MSD, Pfizer, and Gilead Sciences. All the other authors have no conflicts of interest to disclose.

## References

[1] Engert A Balduini C Brand A. The European Hematology Association Roadmap for European Hematology Research: a consensus document. Haematologica. 2016;101:115–208. 26819058 10.3324/haematol.2015.136739 PMC4938336

[2] Ljungman P Schmitt M Marty FM. A mortality analysis of letermovir prophylaxis for cytomegalovirus (CMV) in CMV-seropositive recipients of allogeneic hematopoietic-cell transplantation. Clin Infect Dis. 2019;70:1525–1533. 10.1093/cid/ciz490 PMC7146004 31179485

[3] Marty FM Ljungman P Chemaly RF. Letermovir prophylaxis for cytomegalovirus in hematopoietic-cell transplantation. N Engl J Med. 2017;377:2433–2444. 29211658 10.1056/NEJMoa1706640

[4] Matthes-Martin S Feuchtinger T Shaw PJ. European guidelines for diagnosis and treatment of adenovirus infection in leukemia and stem cell transplantation: summary of ECIL-4 (2011). Transpl Infect Dis. 2012;14:555–563. 23146063 10.1111/tid.12022

[5] Hiwarkar P Kosulin K Cesaro S. Management of adenovirus infection in patients after haematopoietic stem cell transplantation: state-of-the-art and real-life current approach: a position statement on behalf of the Infectious Diseases Working Party of the European Society of Blood and Marrow Transplantation. Rev Med Virol. 2018;28:e1980. 29663594 10.1002/rmv.1980

[6] Cesaro S Dalianis T Hanssen Rinaldo C. ECIL guidelines for the prevention, diagnosis and treatment of BK polyomavirus-associated haemorrhagic cystitis in haematopoietic stem cell transplant recipients. J Antimicrob Chemother. 2018;73:12–21. 29190347 10.1093/jac/dkx324

[7] Ison MG Hirsch HH. Community-acquired respiratory viruses in transplant patients: diversity, impact, unmet clinical needs. Clin Microbiol Rev. 2019;32:e00042–e00019. 10.1128/CMR.00042-19 PMC7399564 31511250

[8] Cordonnier C Einarsdottir S Cesaro S. Vaccination of haemopoietic stem cell transplant recipients: guidelines of the 2017 European Conference on Infections in Leukaemia (ECIL 7). Lancet Infect Dis. 2019;19:e200–e212. 30744963 10.1016/S1473-3099(18)30600-5

[9] Satlin MJ Jenkins SG Walsh TJ. The global challenge of carbapenem-resistant Enterobacteriaceae in transplant recipients and patients with hematologic malignancies. Clin Infect Dis. 2014;58:1274–1283. 24463280 10.1093/cid/ciu052 PMC4038783

[10] Lehners N Schnitzler P Geis S. Risk factors and containment of respiratory syncytial virus outbreak in a hematology and transplant unit. Bone Marrow Transplant. 2013;48:1548–1553. 23811816 10.1038/bmt.2013.94

[11] Eckmanns T Rüden H Gastmeier P. The influence of high-efficiency particulate air filtration on mortality and fungal infection among highly immunosuppressed patients: a systematic review. J Infect Dis. 2006;193:1408–1418. 16619189 10.1086/503435

[12] Iida N Dzutsev A Stewart CA. Commensal bacteria control cancer response to therapy by modulating the tumor microenvironment. Science. 2013;342:967–970. 24264989 10.1126/science.1240527 PMC6709532

[13] Taur Y Jenq RR Perales MA. The effects of intestinal tract bacterial diversity on mortality following allogeneic hematopoietic stem cell transplantation. Blood. 2014;124:1174–1182. 24939656 10.1182/blood-2014-02-554725 PMC4133489

[14] Pierrard J Seront E. Impact of the gut microbiome on immune checkpoint inhibitor efficacy-a systematic review. Curr Oncol. 2019;26:395–403. 31896938 10.3747/co.26.5177 PMC6927774

[15] Sorbara MT Dubin K Littmann ER. Inhibiting antibiotic-resistant Enterobacteriaceae by microbiota-mediated intracellular acidification. J Exp Med. 2019;216:84–98. 30563917 10.1084/jem.20181639 PMC6314524

[16] Haak BW Prescott HC Wiersinga WJ. Therapeutic potential of the gut microbiota in the prevention and treatment of sepsis. Front Immunol. 2018;9:2042. 30250472 10.3389/fimmu.2018.02042 PMC6139316

[17] Fernández-Ruiz M Meije Y Manuel O. ESCMID Study Group for Infections in Compromised Hosts (ESGICH) Consensus Document on the safety of targeted and biological therapies: an infectious diseases perspective (Introduction). Clin Microbiol Infect. 2018;24(suppl 2):S2–S9. 29427801 10.1016/j.cmi.2018.01.029

[18] Maschmeyer G De Greef J Mellinghoff SC. Infections associated with immunotherapeutic and molecular targeted agents in hematology and oncology. A position paper by the European Conference on Infections in Leukemia (ECIL). Leukemia. 2019;33:844–862. 30700842 10.1038/s41375-019-0388-x PMC6484704

[19] Donnelly JP Chen SC Kauffman CA. Revision and update of the consensus definitions of invasive fungal disease from the European Organization for Research and Treatment of Cancer and the Mycoses Study Group Education and Research Consortium. Clin Infect Dis. 2020;71:1367–1376. 31802125 10.1093/cid/ciz1008 PMC7486838

[20] Mikulska M Cesaro S de Lavallade H. Vaccination of patients with haematological malignancies who did not have transplantations: guidelines from the 2017 European Conference on Infections in Leukaemia (ECIL 7). Lancet Infect Dis. 2019;19:e188–e199. 30744964 10.1016/S1473-3099(18)30601-7

[21] Tangye SG Al-Herz W Bousfiha A. Human inborn errors of immunity: 2019 update on the classification from the International Union of Immunological Societies Expert Committee. J Clin Immunol. 2020;40:24–64. 31953710 10.1007/s10875-019-00737-x PMC7082301

[22] Meyts I Bucciol G Quinti I. Coronavirus disease 2019 in patients with inborn errors of immunity: an international study. J Allergy Clin Immunol. 2020;147:520–531. 32980424 10.1016/j.jaci.2020.09.010 PMC7832563

[23] Bastard P Rosen LB Zhang Q. Autoantibodies against type I IFNs in patients with life-threatening COVID-19. Science. 2020;370:eabd4585. 32972996 10.1126/science.abd4585 PMC7857397

[24] Le Clech L Talarmin JP Couturier MA. Early discontinuation of empirical antibacterial therapy in febrile neutropenia: the ANTIBIOSTOP study. Infect Dis (Lond). 2018;50:539–549. 29451055 10.1080/23744235.2018.1438649

[25] Sepriano A Kerschbaumer A Smolen JS. Safety of synthetic and biological DMARDs: a systematic literature review informing the 2019 update of the EULAR recommendations for the management of rheumatoid arthritis. Ann Rheum Dis. 2020;79:760–770. 32033941 10.1136/annrheumdis-2019-216653

[26] Garlanda C Bottazzi B Bastone A. Pentraxins at the crossroads between innate immunity, inflammation, matrix deposition, and female fertility. Annu Rev Immunol. 2005;23:337–366. 15771574 10.1146/annurev.immunol.23.021704.115756

[27] Garlanda C Hirsch E Bozza S. Non-redundant role of the long pentraxin PTX3 in anti-fungal innate immune response. Nature. 2002;420:182–186. 12432394 10.1038/nature01195

[28] Salvatori G Campo S. Current understanding of PTX3 protective activity on Aspergillus fumigatus infection. Med Mycol. 2012;50:225–233. 22309253 10.3109/13693786.2011.648215

[29] Cunha C Aversa F Lacerda JF. Genetic PTX3 deficiency and aspergillosis in stem-cell transplantation. N Engl J Med. 2014;370:421–432. 24476432 10.1056/NEJMoa1211161

[30] Wójtowicz A Lecompte TD Bibert S. PTX3 polymorphisms and invasive mold infections after solid organ transplant. Clin Infect Dis. 2015;61:619–622. 25977268 10.1093/cid/civ386

[31] Fisher CE Hohl TM Fan W. Validation of single nucleotide polymorphisms in invasive aspergillosis following hematopoietic cell transplantation. Blood. 2017;129:2693–2701. 28270451 10.1182/blood-2016-10-743294 PMC5428460

[32] Brunel AS Wójtowicz A Lamoth F. Pentraxin-3 polymorphisms and invasive mold infections in acute leukemia patients receiving intensive chemotherapy. Haematologica. 2018;103:e527–e530. 29880612 10.3324/haematol.2018.195453 PMC6278985

[33] Lamoth F Rubino I Bochud PY. Immunogenetics of invasive aspergillosis. Med Mycol. 2011;49(suppl 1):S125–S136. 20840014 10.3109/13693786.2010.516408

[34] Plantinga TS van der Velden WJ Ferwerda B. Early stop polymorphism in human DECTIN-1 is associated with increased candida colonization in hematopoietic stem cell transplant recipients. Clin Infect Dis. 2009;49:724–732. 19614557 10.1086/604714

[35] Cunha C Di Ianni M Bozza S. Dectin-1 Y238X polymorphism associates with susceptibility to invasive aspergillosis in hematopoietic transplantation through impairment of both recipient- and donor-dependent mechanisms of antifungal immunity. Blood. 2010;116:5394–5402. 20807886 10.1182/blood-2010-04-279307

[36] Hill JA Li D Hay KA. Infectious complications of CD19-targeted chimeric antigen receptor-modified T-cell immunotherapy. Blood. 2018;131:121–130. 29038338 10.1182/blood-2017-07-793760 PMC5755046

[37] Park JH Romero FA Taur Y. Cytokine release syndrome grade as a predictive marker for infections in patients with relapsed or refractory B-cell acute lymphoblastic leukemia treated with chimeric antigen receptor T cells. Clin Infect Dis. 2018;67:533–540. 29481659 10.1093/cid/ciy152 PMC6070095

[38] Lee DW Kochenderfer JN Stetler-Stevenson M. T cells expressing CD19 chimeric antigen receptors for acute lymphoblastic leukaemia in children and young adults: a phase 1 dose-escalation trial. Lancet. 2015;385:517–528. 25319501 10.1016/S0140-6736(14)61403-3 PMC7065359

[39] Schuster SJ Bishop MR Tam CS. Tisagenlecleucel in adult relapsed or refractory diffuse large B-cell lymphoma. N Engl J Med. 2019;380:45–56. 30501490 10.1056/NEJMoa1804980

[40] Hill JA Giralt S Torgerson TR. CAR-T - and a side order of IgG, to go? - Immunoglobulin replacement in patients receiving CAR-T cell therapy. Blood Rev. 2019;38:100596. 31416717 10.1016/j.blre.2019.100596 PMC6810871

[41] Cordeiro A Bezerra ED Hirayama AV. Late events after treatment with CD19-targeted chimeric antigen receptor modified T cells. Biol Blood Marrow Transplant. 2020;26:26–33. 31419568 10.1016/j.bbmt.2019.08.003 PMC6953906

[42] Hill JA Krantz EM Hay KA. Durable preservation of antiviral antibodies after CD19-directed chimeric antigen receptor T-cell immunotherapy. Blood Adv. 2019;3:3590–3601. 31743392 10.1182/bloodadvances.2019000717 PMC6880890

[43] Seif M Einsele H Löffler J. CAR T cells beyond cancer: hope for immunomodulatory therapy of infectious diseases. Front Immunol. 2019;10:2711. 31824500 10.3389/fimmu.2019.02711 PMC6881243

[44] Yakoub-Agha I Chabannon C Bader P. Management of adults and children undergoing chimeric antigen receptor T-cell therapy: best practice recommendations of the European Society for Blood and Marrow Transplantation (EBMT) and the Joint Accreditation Committee of ISCT and EBMT (JACIE). Haematologica. 2020;105:297–316. 31753925 10.3324/haematol.2019.229781 PMC7012497

